# Stepped Care, System Architecture and Mental Health Services in Australia

**DOI:** 10.5334/ijic.2505

**Published:** 2016-09-14

**Authors:** David Perkins

**Affiliations:** 1 University of Newcastle, AU

**Keywords:** mental health, system architecture, stepped care, integrated mental health

In its Review of Mental Health Programmes and Services (2014) the Australian National Mental Health Commission (MHC) concluded:

On the basis of our findings, it is clear that the mental health system has fundamental structural shortcomings. This same conclusion has been reached by numerous other independent and governmental reviews.

…

*The overall impact of a poorly planned and badly integrated system is a massive drain on peoples’ wellbeing and participation in the community – on jobs, on families and on Australia’s productivity and economic growth.* [1]

The report was published under the banner “contributing lives, thriving communities” which summarises the mission and values of the MHC. It concluded that three key components of change were required: people centred design principles, a new system architecture, and a shift of funding to more efficient and effective ‘upstream’ services and supports [1]. Part of the Australian Government’s response to this report was to establish a regional planning process to address these three components to be led by 31 Primary Health Networks which cover the whole of Australia [2].

Mental health systems must balance the challenges of caring for people with mental illnesses and helping to prevent mental illness at individual, family and community levels. These objectives can require very different approaches and are often the responsibility of different organisations and disciplines.

On the basis of my research experience in mental health systems and services I propose a minimalist model of stepped care as a contribution to this integrated systems architecture recommended by the MHC [3,4,5,6,7,8].

## Mental health care in Australia

The Australian mental illness system includes public, private and voluntary sector providers, multiple professions and disciplines, funded by different levels of government, and importantly it includes consumers, family members and friends acting as carers.

Some providers specialise in mental illness care, others including general practitioners care for many patients with mental as well as physical health problems. Others may not see themselves as meeting the needs of those with mental health problems include housing, education and employment providers, aged and childcare services, and many working in the disability and welfare sectors.

This system has developed incrementally over many years during which time treatment options have expanded and it is in need of reform [1]. It has been suggested that this system focuses on the needs of providers rather than patients, is slow to prevent mental health problems and intervene early and that the financial payments for providers incentivise activities rather than outcomes. It is not surprising that a system which has developed over many years in an incremental fashion might need attention if it is to incorporate new knowledge and deal effectively with changing needs.

More importantly, evidence shows that many people who could benefit from mental health care do not access services [9]. Many of those with severe and enduring mental health problems also have substance abuse and comorbid physical health problems, and their life expectancy can be reduced by as much as 15 years compared with the general population [10]. These people also suffer severely impaired quality of life and require high levels of support from carers and service providers.

The services available in different localities vary for a number of reasons. Workforce distribution does not always match needs. Rural and remote communities may experience skills shortages among specialist public and private sector providers. Special needs such as experienced in Aboriginal and Torres Strait Islander, culturally and linguistically diverse (CALD) and low socioeconomic status communities may need particular investments in care and treatment.

These problems are neither new nor unrecognised and a number of attempts have been made to better integrate care. Between 2000–2003 there were three National Mental Health Integration Projects in Australia designed to better integrate public and private specialist mental health services and there have been many such projects since [11].

The “Better Access” initiative 2006-onwards was designed to improve access to mental health specialists (psychologists and psychiatrists) in the community. It requires the preparation of a mental health plan for a patient by a general practitioner to ensure that specialist care is closely aligned with ongoing GP care [12]. The Headspace programme providing co-located mental health services for children and young people started in 2010 [13]. In 2012 a “Partners in Recovery” (PIR) program was introduced to build multi-provider, individualised service packages for those in the community who have not responded to normal care [14]. In 2016 the “National Disability Insurance Scheme” scheme is being implemented nationally to provide individually tailored care packages for those with enduring disabilities living in the community [15].

Each of these initiatives are well-intentioned incremental attempts to address important problems such as adolescent mental health and the early onset of mental illness but collectively they contribute to a disjointed service.

The creation in 2015 of Primary Health Networks (PHNs) sharing boundaries with one or more Local Hospital Networks has provided an opportunity to plan community services at a regional level, assess the needs of communities, the gaps in current services and commission to improve local mental health services.

## The need for a stepped care approach

**Figure 1:**
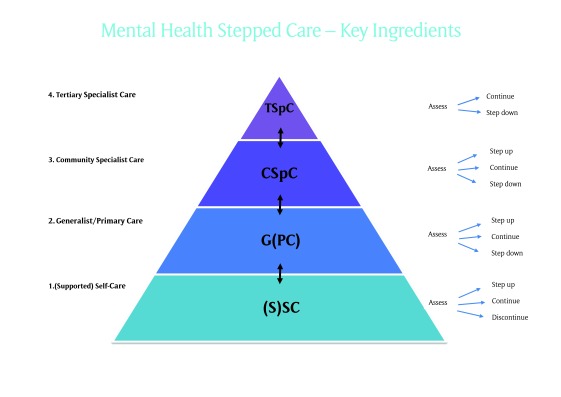
A simple model of stepped care.

If the Mental Health Commission changes are to be implemented and more importantly if the individuals and communities are to benefit from efficient and effective services, then the Primary Health Networks must focus on the system architecture. I suggest that this might be achieved through the adoption of a stepped care framework (see figure 1).

It should be noted that due to the different needs, resources and services of particular localities and communities, no single model will suit all circumstances.

A simple model of stepped care is proposed which can be elaborated upon to meet particular needs and to take account of high resource (rich) communities and low resource (poor) communities.

The model I am proposing includes four steps:

1. *Self-care with or without support* might be web-based, use workbooks or similar materials, and may or may not be monitored by a health professional.
2. *Community-based care* provided by a general practitioner or other generalist clinician- might include psychological and/or pharmaceutical therapies.
3. *Community-based specialist care* is usually provided by a community based mental health clinician or team, and
4. *Tertiary specialist care* will continue to be needed but as the exception rather than the norm.

Two principles apply. Firstly, all care should incorporate evidence-based treatments and staff should be appropriately skilled. This might require an investment in training for any clinicians providing mental health care. Secondly, regular patient assessment should enable prompt decisions to escalate care to a higher intensity (step-up), to continue treatment or to reduce the intensity of care (step-down). Thus, a GP might encourage a patient to try a programme of supported self-care using a web-based treatment, recognising that timely escalation to a more appropriate level (step-up) may be needed if the patient does not respond to the treatment.

This model is designed to apply to rich service environments such as capital cities where there may be a range of intermediate steps and also to lean environments such as rural and remote communities where specialist care may be provided by visiting services and by teleconference technologies. It does, perhaps optimistically, assume that near universal Internet coverage by cable or satellite is within sight.

Some staff in generalist, primary care and NGO settings will feel ill prepared to operate in an integrated, stepped care environment. Opportunities should be made available so that they can obtain appropriate education and skilled support or supervision in their developing roles.

Financial reimbursements must be aligned to service models and may need to be adjusted over time so that activities such as case-conferencing and supporting other staff are compensated as core business and not regarded as a unpaid chore and avoided.

Most importantly, PHNs will need to achieve a measure of normative agreement about an appropriate model of care and I suggest that this simple model provides a starting point. Many requiring specialist care whether within the community or in tertiary services will also require services from education, employment, housing, welfare and other providers which will need to be integrated appropriately but that is the subject of another paper.

Finally, integration will need to be progressive and introduced over an appropriate period. It is not possible that all Primary Health Networks and their partners will progress at the same pace. Some may demonstrate solid progress and warrant increased commissioning autonomy while others may benefit from support by PHNs that have made exceptional progress.

## Author Information

David Perkins is Professor of Rural Health Research and Director of the Centre for Rural and Remote Mental Health, University of Newcastle, Orange NSW Australia. He has worked as a senior manager in the UK NHS and in Universities in the UK and Australia. He is an editor of IJIC, a Board Member of IFIC and the coordinator of IFIC Australia.
